# Elevated Risk of Endometrial Cancer and Precursor Lesions in Patients With Myotonic Dystrophy: A Retrospective Study at a Single Institution in Japan

**DOI:** 10.1111/jog.70221

**Published:** 2026-02-26

**Authors:** Ruka Hano, Hirofumi Ando, Marina Fujioka, Masako Nakajima, Manaka Shinagawa, Hodaka Takeuchi, Mitsuko Shinagawa, Hisanori Kobara, Tsuneaki Yoshinaga, Tsutomu Miyamoto

**Affiliations:** ^1^ Department of Obstetrics and Gynecology Shinshu University School of Medicine Nagano Japan; ^2^ Department of Medicine (Neurology and Rheumatology) Shinshu University School of Medicine Nagano Japan

**Keywords:** atypical endometrial hyperplasia, endometrial cancer, myotonic dystrophy

## Abstract

**Aim:**

Myotonic dystrophy (MD) has been associated with an increased risk of endometrial cancer (EC) in Western countries; however, data from Japan are limited. This study aimed to evaluate the incidence and clinical characteristics of EC and its precursor lesion, atypical endometrial hyperplasia (AEH), in Japanese patients with MD.

**Methods:**

We retrospectively reviewed medical records of female patients with MD (MD group, *n* = 36) and those with other types of muscular dystrophy (control group, *n* = 84) treated at a single institution between 2008 and 2023.

**Results:**

EC/AEH was identified in 5 of 36 patients in the MD group (13.9%), including 3 cases of EC and 2 of AEH, whereas 1 case of EC (1.2%) was observed among 84 control patients, indicating a significantly higher incidence in the MD group (*p* = 0.014). The median age at diagnosis of EC/AEH in the MD group was 45 years, which was significantly younger than that of non‐MD EC/AEH cases treated during the same period (median 59 years). All surgically treated EC cases were early‐stage endometrioid carcinoma and achieved favorable oncological outcomes, although perioperative respiratory complications were observed.

**Conclusions:**

Japanese MD patients have a significantly higher risk of developing EC/AEH at a younger age compared with non‐MD patients. These findings highlight the importance of regular gynecological surveillance in women with MD.

**Abbreviations**
AEH: atypical endometrial hyperplasia
BMI: body mass index
EC: endometrial cancer
MD: myotonic dystrophy

## 1 Introduction

Myotonic dystrophy (MD) is the most common muscular dystrophy in adults and is an autosomal dominant disorder caused by repeat expansions in specific genes: Type 1 is due to a CTG trinucleotide repeat in the *DMPK* gene, and Type 2 results from a CCTG tetranucleotide repeat in the *CNBP* (*ZNF9*) gene [1, 2]. The prevalence of MD was previously estimated to be 9.99 (95% CI: 5.62–15.53) per 100 000 cases [3]; however, a recent study on newborn screening indicated that the incidence of the *DMPK* gene with CTG repeat expansions ≥ 50 was 4.76 per 10 000 births (95% confidence interval 2.86–6.67), suggesting a markedly higher incidence than previously considered [4]. These genetic mutations lead to progressive muscle weakness and myotonia, along with multi‐systemic involvement, including cataracts, cardiac conduction abnormalities, infertility, and insulin resistance [5]. There is currently no curative treatment for MD, and only management for complications and symptoms is conducted. MD patients often require artificial respiration at approximately 50 years of age, and due to respiratory infections or treatment failure and cardiac conditions, such as arrhythmia, the average life expectancy is approximately 54 years [6, 7].

Cases of malignant tumors complicating MD have sporadically been reported [8], leading to the first large‐scale study in Scandinavia in 2011, which showed a high incidence of malignancies, including endometrial cancer (EC) [9]. Subsequent studies in various countries and regions have consistently demonstrated a significantly higher incidence of malignancies in MD patients. Although the types of malignancies with a higher incidence than in the general population vary across studies, a significant increase in the incidence of EC has consistently been reported.

Park et al. investigated the comorbidity of malignant tumors in MD patients using South Korea's national health insurance database and reported that the distribution of cancer types appeared to differ from that in the general population [10]. However, differences in the incidence of malignancies between MD patients and other populations have not been reported in Eastern countries, including Japan. Therefore, we investigated the prevalence of EC and its precancerous lesion, atypical endometrial hyperplasia (AEH), in MD cases at our institution.

## 2 Materials and Methods

### 2.1 Patients

Thirty‐six female patients with MD (MD cohort) and 84 female patients with other types of muscular dystrophy (Control cohort) who visited Shinshu University Hospital between 2008 and 2023 were retrospectively reviewed. The cases included in the MD cohort were confirmed to have MD based on clinical symptoms, a family history, and genetic testing. The control cohort (*n* = 84) was used to evaluate the risk of cancer in individuals who presumably have similar medical accessibility, comprising other types of muscular dystrophies, for example, Duchenne muscular dystrophy, Becker muscular dystrophy, and limb‐girdle muscular dystrophy (Table 1). Patient data, including age, the activities of daily living, body mass index (BMI), comorbidities, pathological diagnosis, and family history, were collected from medical records. Cases diagnosed with EC/AEH prior to the observation period and cases who had undergone hysterectomy due to other diseases were excluded. End of the follow‐up was defined as: death, transfer, last visit, or conduction of hysterectomy. In addition, 996 EC/AEH cases that were treated in the hospital in the same period who did not have concomitant MD were retrospectively reviewed. Age at the diagnosis of EC/AEH was collected from medical records.

**TABLE 1:** Types of muscular dystrophy comprising the control cohort.

	*n* (%)
Duchenne muscular dystrophy	31 (36.9%)
Facioscapulohumeral muscular dystrophy	18 (21.4%)
Limb‐girdle muscular dystrophy	11 (13.1%)
Becker muscular dystrophy	8 (9.5%)
Oculopharyngeal Muscular Dystrophy	5 (6%)
Fukuyama type congenital muscular dystrophy	3 (3.6%)
Others	8 (9.5%)

### 2.2 Statistical Analysis

Continuous variables were presented as the median and interquartile range and were analyzed using the Mann–Whitney U test. Categorical variables were analyzed using Fisher's exact test. In all analyses, the significance of differences was defined as a *p*‐value < 0.05. All statistical analyses were performed with EZR (Saitama Medical Center, Jichi Medical University, Saitama, Japan) [11], which is a graphical user interface of R (The R Foundation for Statistical Computing, Vienna, Austria).

## 3 Results

The demographics of the MD and control cohorts are shown in Table 2. There were no significant differences in age or the prevalence of diabetes mellitus between the MD and control cohorts. Although a significant difference was observed in BMI, the percentage of overweight cases with a BMI ≥ 25, a risk factor for the development of EC/AEH [12], did not significantly differ. The median period of observation in the MD cohort was 49.5 months [IQR 6.4, 98.8], which was not significantly different from that in the control cohort (median 52.6 months [IQR 5.3, 120.0], *p* = 0.611).

**TABLE 2:** Demographics of MD and control cohorts.

	MD cohort	Control cohort	
	(*n* = 36)	(*n* = 84)	*p*
Age (median [IQR])	47.5 [41.5, 55.25]	48 [35.8, 63.3]	0.66
Diabetes (%)	1 (2.8%)	1 (1.2%)	0.512
BMI (median [IQR])	22.1 [20.9, 25.6]	20.3 [17.6, 22.7]	0.04
BMI ≥ 25 (%)	8 (22.2%)	17 (20.2%)	0.798
Nulliparous	13/30 (43.3%)	24/74 (32.4%)	0.367
Menstrual irregularity	8/15 (53.3%)	2/5 (28.6%)	0.381
Observation period (median [IQR])	46.4 [7.6, 111]	54.2 [7.0, 100.1]	0.786
Gynecological checkups during observation period (≥ 1 time)	10 (27.8%)	10 (11.9%)	0.058

Abbreviations: BMI, body mass index; IQR, interquartile range.

**FIGURE 1:**
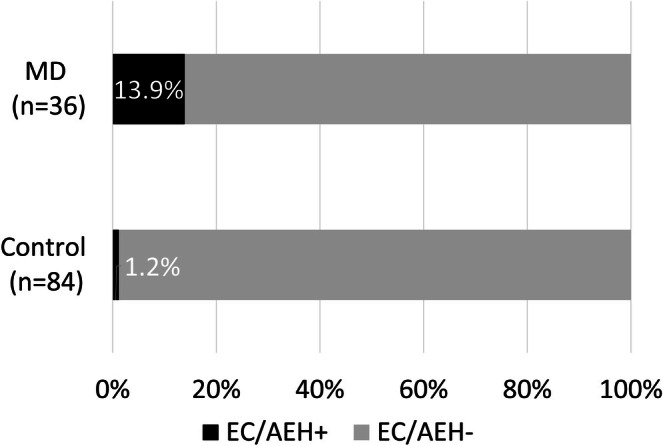
The incidence proportion (risk) of EC/AEH in the MD and control cohortsMD cohort showed significant higher odds to develop EC/AEH than control cohort.

**FIGURE 2:**
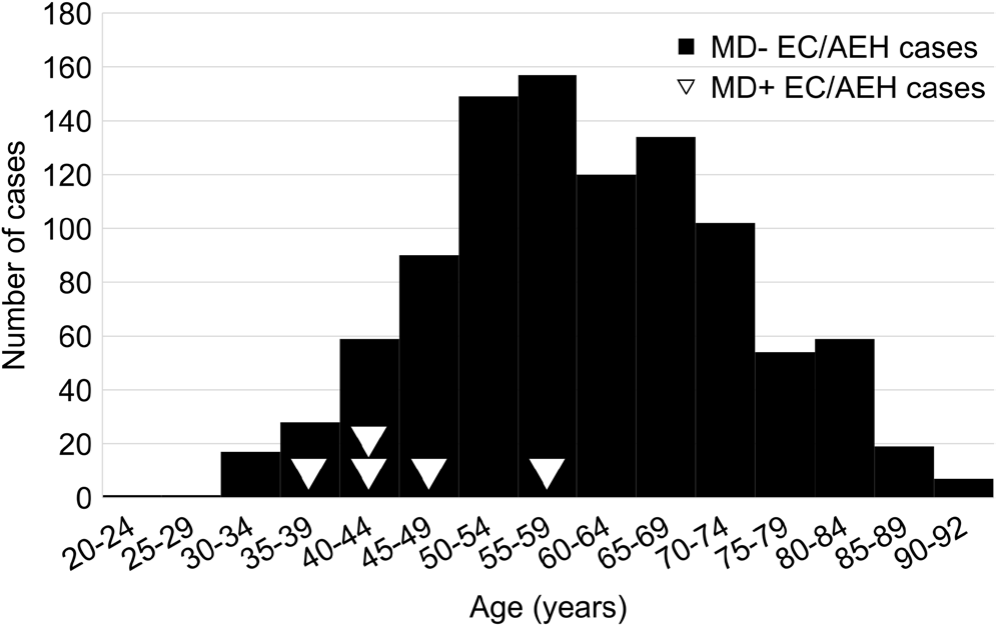
Histogram of the age distribution of non‐MD EC/AEH cases and EC/AEH cases with MD.The onset of EC/AEH was significantly younger in MD cases than in those without MD.

Among 36 female MD patients, there were 3 cases of EC and 2 of AEH, whereas among 84 patients with other muscular dystrophy, there was only 1 case of EC. The incidence proportion (risk) of EC/AEH in the MD and control cohorts was 13.9% and 1.2%, respectively. The MD cohort showed a significantly higher odds of the development of EC/AEH compared with the control cohort, with an odds ratio of 13.1 (95% confidence interval 1.39–638.2, *p* = 0.0092) (Figure 1). Median age at the diagnosis of EC/AEH in the MD cohort was 45 years. In contrast, median age at the diagnosis of 996 cases of EC/AEH during the same period was 59 years. A significant difference in age was observed between the MD group and the non‐MD group (Wilcoxon rank sum test, *W* = 841.5, *p*‐value = 0.01057). The median difference was −14.0 years (95% confidence interval: −25.0 to −4.0 years) (Figure 2).

The backgrounds and clinical courses of the three cases of EC with MD and two cases of AEH are shown in Table 3. All cases were diagnosed with or strongly suspected to be MD Type 1. Cases #1, #2, and #3 showed elevated CTG repeat numbers within the *DMPK* gene by genetic diagnosis. Cases #4 and #5 were diagnosed based on family history and clinical symptoms. Regarding the diagnosis of EC/AEH, endometrial thickening was discovered in two cases during gynecological visits for other conditions, such as cervical intraepithelial neoplasia; endometrial thickening was noted in another two cases during routine check‐ups, and one case presented with abnormal genital bleeding. There were three cases in which the age at onset was 45 years or younger. Three cases had a BMI ≥ 25 and two cases had diabetes mellitus. In addition, four cases had menstrual irregularities, and three had never given birth, suggesting that they were at risk of developing EC. Restrictive ventilatory impairment was observed in all cases. In three cases, the activities of daily living were not impaired, while assistance was required for daily activities in two cases (Case #1 and #5).

**TABLE 3:** Backgrounds and clinical courses of EC/AEH cases with MD.

Case #	Age	BMI	comorbidity	Family history of MD	Menstruation cycle	Gravidity and parity	Type of MD (CTG repeat numbers)	Activities of daily living
1	58	20.6	Diabetes mellitus, hypertension	Father	Regular	G0	Type 1 (800–900)	In a wheelchair
2	41	29	CIN3, hepatic steatosis	2 children	Irregular	G3P3	Type 1 (600)	Independent
3	44	28.2	None	2 siblings and a niece	Irregular	G0	Type 1 (1000)	Independent
4	38	22	Diabetes mellitus, hepatic steatosis	A cousin	Irregular	G2P0SA2	Type 1 (NA)	Independent
5	49	25	Hepatic steatosis	2 siblings and a child	Irregular	G2P1	Type 1 (NA)	Bedridden

Case #	Treatments	FIGO stage	Histological type	Complication of treatment	Oncological outcome (observation period)
1	TAH + BSO	IB	Endometrioid Grade 1	None	NED (1.5 years)
2	TLH + BSO + PLA	IA	Endometrioid Grade 1	Deterioration of respiratory function and introduction of home oxygen therapy	NED (3.4 years)
3	TLH + BSO	IA	Endometrioid Grade 1	Pneumothorax	NED (6.3 years)
4	TLH + BSO		AEH	Transient respiratory failure	NED (8.2 years)
5	Observation		AEH	NA	AWD (1.3 years)

Abbreviations: AEH, atypical endometrial hyperplasia; AWD, alive with disease; BSO, bilateral salpingo‐oophorectomy; CIN, cervical intraepithelial neoplasia; FIGO, International Federation of Gynecology and Obstetrics; MD, myotonic dystrophy; NA, not available; NED, no evidence of disease; PLA, pelvic lymphadenectomy; TAH, total abdominal hysterectomy; TLH, total laparoscopic hysterectomy.

Regarding the treatment of the five cases of EC/AEH, curative surgery was performed on four. In the remaining case, respiratory function suddenly deteriorated during the examination and treatment in our department was not possible; therefore, follow‐up observations were continued at a local hospital. Several respiratory‐related issues were recorded as incidents related to surgery. In Case #2, hypoxemia due to respiratory muscle dysfunction persisted during the postoperative period, requiring the initiation of home oxygen therapy. In case #3, pneumothorax recurred after surgery, requiring chest drainage. In Case #4, temporary hypoxemia developed after surgery. The International Federation of Gynecology and Obstetrics 2008 staging of three cases of EC was IB in one case and IA in two cases, and the histological type was endometrioid carcinoma Grade 1 in all cases. All four cases that underwent surgery have survived without recurrence. In addition, no cases of malignancy other than EC/AEH were observed in any of the cases.

## 4 Discussion

This is the first study to examine the incidence proportion (risk) of EC/AEH in MD patients in Japan. The results obtained revealed that MD patients were more susceptible to developing EC/AEH than control cases with similar medical accessibility. Furthermore, EC/AEH generally developed at a younger age in MD patients.

The present study showed that the rate of developing EC/AEH was higher in the MD cohort than in the control cohort. Previous studies reported the incidence of malignancies in MD patients [9, 13, 14, 15, 16, 17, 18, 19]. The regions where these studies were conducted included the United States, the United Kingdom, Spain, Sweden, and Denmark. Comparison subjects in these studies varied, including comparisons with the general population or patients with muscular dystrophies other than MD. All studies indicated a higher incidence of cancer overall in MD patients. Furthermore, most studies showed a significantly higher incidence of EC in MD patients [9, 14, 15, 16, 17, 18, 19]. Regarding malignancies other than EC, the incidence of ovarian cancer, thyroid cancer, testicular cancer, colorectal cancer, and skin tumors was significantly higher in MD patients. This was a small‐scale study that lacked sufficient statistical power for the detection of tumors other than EC/AEH. However, the result showing the high incidence of EC/AEH supports findings from other regions and suggests that a similar pattern exists in Eastern countries. Although a direct comparison with this cohort was not possible, since the lifetime prevalence of EC in Japan is 2.1% [20], its rate is generally considered to be higher in MD patients than in the general population. Therefore, nationwide studies that examine the relationship between tumors, including EC/AEH, and MD are warranted.

The present results also suggest that EC/AEH generally develops at a younger age in MD patients than in control cases. Gadalla et al. reported that the ratio of gynecological cancers in MD patients to the general population in the United States was significantly higher in those aged < 50 years than in those aged ≥ 50 years (standardized proportion ratios, 2.4 vs. 1.1 for age < 50 vs. ≥ 50 years, respectively; *p* for heterogeneity = 0.004) [19]; though this study did not specify the type of gynecological cancer. In addition, Alsaggaf et al. found that benign tumors, including colorectal polyps, benign tumors of the nervous system, and thyroid nodules, were detected more frequently and at a younger age in Type 1 MD patients than in the general population [21]. In consideration of the tumorigenic potential of MD, tumors such as EC/AEH may develop at a younger age than in the general population.

The biological mechanisms underlying the increased incidence of cancer in MD patients remain unclear; however, several theories have been proposed. The introns of the *DMPK* and *CNBP* (*ZNF9*) genes are extended by repeat sequences, resulting in abnormally long transcripts that include these repeat sequences. This RNA accumulates in the nucleus and changes the function of RNA splicing factors, such as MBNL1 and the CELF family of RNA‐binding proteins. Changes in the functional levels of these proteins cause splicing abnormalities in various target transcripts. These targets include cardiac troponin T, insulin receptor, muscle chloride Channel 1, and microtubule‐associated tau, leading to the characteristic symptoms of MD, such as muscle weakness and cardiac conduction defects. A similar phenomenon may also be involved in tumorigenesis [8, 22]. Another example of the mechanism linking MD and carcinogenesis is a study showing differences in the expression levels of multiple cancer‐related genes, including PDK4, DAPK1, CASP5, and PLA2G7, in DM1 patients, and a reduction in tumor suppressor microRNA family 200c/141 in female DM1 patients [15]. Several therapies have been preclinically developed, with the aim of specifically degrading or neutralizing mutant RNA to release trapped RNA‐binding proteins and restore normal cellular functions [23]. These therapies target the basic cause of MD and also appear to offer potential effect against tumorigenesis.

The potential molecular mechanisms by which MD increases EC/AEH remain unclear, as is the case with other tumors. Regarding factors that may be risk factors for EC/AEH in MD, some are thought to increase while others are not. First, obesity is known to be increased in MD [24]. On the other hand, hypothalamic amenorrhea is reported to be increased in MD [25], which is thought to cause a low estrogen state; however, to our knowledge, there are still no reports directly investigating whether anovulatory cycles are more common compared to the general population. Furthermore, reports indicate no difference in pregnancy rates compared to the general population [26], and there is no evidence of a higher proportion of nulliparous women. In this report, nulliparity and menstrual irregularity showed no significant differences. However, it cannot be ruled out that the higher BMI in the MD cohort contributed to EC/AEH occurrence. Analysis with matched subjects in a larger‐scale study is warranted.

The limitations of this study are as follows. Being retrospective and having a small number of cases and events, multivariate analysis and propensity score matching could not be performed, meaning confounding factors could not be fully adjusted for. Furthermore, as the control group did not represent the general population, there are limitations to directly extrapolating the results. Nevertheless, despite these limitations, this study offers the first Japanese data suggesting a markedly elevated risk and younger onset of EC/AEH in patients with MD, contributing to the growing international evidence and supporting the need for structured gynecological monitoring in this population.

In conclusion, the incidence of EC and AEH was higher in MD patients than in non‐MD patients. Furthermore, EC and AEH generally developed at a younger age in MD patients. Therefore, it is considered desirable for MD patients to undergo continuous gynecological follow‐ups in collaboration with neurologists.

## Author Contributions

**Ruka Hano:** investigation, writing – original draft. **Hirofumi Ando:** investigation, writing – review and editing, visualization, project administration. **Marina Fujioka:** investigation. **Masako Nakajima:** investigation. **Manaka Shinagawa:** investigation. **Hodaka Takeuchi:** investigation. **Mitsuko Shinagawa:** investigation. **Hisanori Kobara:** investigation. **Tsuneaki Yoshinaga:** conceptualization, data curation, writing – review and editing, supervision. **Tsutomu Miyamoto:** conceptualization, writing – review and editing, supervision.

## Funding

The authors have nothing to report.

## Disclosure

An earlier version of this article was presented at the following conferences: the 77th Annual Congress of the Japan Society of Obstetrics and Gynecology, Okayama, Japan, May 23–25, 2025. The 67th Annual Meeting of Japan Society of Gynecologic Oncology, Tokyo, Japan, July 17–19, 2025.

## Ethics Statement

This study was approved by the Ethics Committee of Shinshu University (Approval Number: 6375).

## Consent

The study was exempt from the need to obtain individual consent by offering subjects the opportunity to opt out because this was a retrospective study with no interventions for patients. We also obtained comprehensive consent for the usage of clinical data for clinical studies at the time of the first visit.

## Conflicts of Interest

Tsutomu Miyamoto is an Editorial Board member of the JOGR and a co‐author of this article. To minimize bias, he was excluded from all editorial decision‐making related to the acceptance of this article for publication. The other authors declare no conflicts of interest.

## Data Availability

The data are not publicly accessible due to privacy and ethical restrictions. Although the raw data analyzed in this study have been anonymized, we did not obtain participant consent for public sharing, and ethics committee approval for public disclosure was not granted. However, the corresponding author may provide access to the data upon a reasonable request, subject to the oversight of the ethics committee.
